# Assessment of Cardiovascular Risk in the PURE Poland Cohort Study Using the Systematic Coronary Risk Evaluation (SCORE) Scale and Galectin-3 Concentrations: A Cross-Sectional Study

**DOI:** 10.3390/ijms26073064

**Published:** 2025-03-27

**Authors:** Adrian Martuszewski, Patrycja Paluszkiewicz, Katarzyna Połtyn-Zaradna, Agnieszka Kusnerż, Rafał Poręba, Andrzej Szuba, Paweł Gać, Katarzyna Zatońska

**Affiliations:** 1 Department of Environmental Health, Occupational Medicine and Epidemiology, Wroclaw Medical University, Mikulicza-Radeckiego 7, 50-345 Wrocław, Poland; adrian.martuszewski@student.umw.edu.pl (A.M.);; 2 Department of Neurology, Specialist Hospital in Walbrzych, Sokołowskiego 4, 58-309 Wałbrzych, Poland; 3 Department of Emergency Medical Service, Wroclaw Medical University, Bartla 5, 50-367 Wrocław, Poland; 4 Department of Population Research and Prevention of Civilization Diseases, Wroclaw Medical University, Bujwida 44, 50-372 Wrocław, Poland; 5 Department of Biological Principles of Physical Activity, Wroclaw University of Health and Sport Sciences, 51-612 Wrocław, Poland; 6 Department of Angiology and Internal Diseases, Wroclaw Medical University, Borowska 213, 50-556 Wrocław, Poland; 7 Centre of Diagnostic Imaging, 4th Military Hospital, Weigla 5, 50-981 Wrocław, Poland

**Keywords:** cardiovascular diseases, risk assessment, galectin-3 level, biomarkers, SCORE scale

## Abstract

The SCORE (Systematic Coronary Risk Evaluation) scale is useful for cardiovascular disease (CVD) risk stratification. However, there is a new biomarker, namely, galectin-3 (gal-3), that offers additional predictive value. This cross-sectional study assessed the relationship between serum gal-3 concentrations and CVD risk based on the SCORE scale in a Polish cohort from the PURE study. A total of 259 participants with complete cholesterol, blood pressure (BP), and smoking data were included. Individuals with myocardial infarction, stroke, diabetes, or renal failure were excluded. Gal-3 concentrations were measured using ELISA, and statistical analyses examined associations with SCORE categories. The median gal-3 concentration was 221.32(161.64–360.00) ng/mL. Higher gal-3 concentrations were found in older individuals, smokers, and those with elevated BP(*p* < 0.05). Positive correlations were observed with SCORE values (r = 0.39) and systolic BP (r = 0.64). Participants with SCORE ≥5% had significantly higher gal-3 concentrations. ROC analysis showed moderate diagnostic value for SCORE ≥ 10% (sensitivity 0.618 and specificity 0.810). Higher SCORE and diastolic BP are independent risk factors for increased galectin-3 concentrations, explaining 79.5% of the variance in the studied group. Serum gal-3 is significantly associated with CVD risk estimated by SCORE, supporting its potential role in risk stratification.

## 1. Introduction

In Europe, cardiovascular diseases (CVDs) constitute the major cause of mortality, contributing to nearly half of all deaths [1].

Effective cardiovascular prevention utilizes tools such as the SCORE (Systematic Coronary Risk Evaluation) scale, which assesses the 10-year likelihood of mortality by considering factors like cholesterol concentrations, gender, age, blood pressure (BP), and tobacco smoking [2]. This scale categorizes 10-year risk as follows: very low (<1%), low (1–4%), moderate (5–9%), and very high (≥10%). SCORE, therefore, is a cornerstone in identifying those individuals who may benefit most from intensive preventive strategies [2].

Biomarkers such as galectin-3 (gal-3), which is produced predominantly by macrophages, have gained attention for their potential to refine cardiovascular risk prediction. Galectin-3 takes part in inflammation, the remodeling of cardiac tissue, and fibrosis [3]. Elevated gal-3 concentrations were observed in heart failure (including that with preserved EF), arterial hypertension, atherosclerosis, and diabetes [4,5], with meta-analyses demonstrating its prognostic value for HF development (HR = 1.32) [6]. Unlike natriuretic peptides (e.g., NT-proBNP), gal-3 reflects active fibrotic and inflammatory processes, suggesting its complementary role in risk stratification [7]. Gal-3 is acknowledged by regulatory bodies, including the FDA, as a prognostic parameter for chronic HF and is also emphasized in guidelines from the AHA and the ESC. However, its broader utility in cardiovascular risk assessment, particularly in comparison to the SCORE scale, remains underexplored [8,9,10].

The Polish subpopulation in the PURE (Prospective Urban and Rural Epidemiological) study provides a unique opportunity for cardiovascular risk research due to its comprehensive longitudinal design. It offers detailed information on modifiable and non-modifiable risk factors in both rural and urban settings of the Lower Silesia region. This relatively underexplored subpopulation enables comparative analyses and a deeper understanding of cardiovascular health determinants in a specific geographic and demographic environment [11].

The objective of our cross-sectional study was to assess the association between serum gal-3 concentrations and CVD risk, as determined by the SCORE scale, in a Polish cohort from the PURE study.

The confirmation of gal-3’s additional predictive value may provide further insight into its clinical utility and support the implementation of routine gal-3 measurement to optimize CVD prevention.

## 2. Results

We wanted to evaluate the relationship between gal-3 levels and CVD risk, as determined by the SCORE scale. The analysis did not include direct clinical incidents, e.g., myocardial infarction and stroke. The participants had an average age of 55.54 (8.00) years (42.1% male). Table 1 summarizes quantitative and qualitative characterization of the patient cohort. The mean SCORE value was 2.96 (3.33). The distribution of participants across the SCORE categories was as follows: 14.3% of participants had a SCORE < 1%, 64.5% were in the SCORE 1–4% category, 13.1% fell into the SCORE 5–9% range, and 8.1% had a SCORE ≥ 10%. The median galectin-3 concentration was 221.32 (161.64–360.00) ng/mL.

Table 2 presents serum gal-3 concentrations in subgroups determined based on the SCORE questionnaire. Gal-3 concentrations were significantly higher in older participants. Similarly, males exhibited higher concentrations of gal-3 than females (*p* < 0.05), and gal-3 concentrations were also increased in smokers compared to non-smokers (*p* < 0.05). Participants with elevated systolic blood pressure also had higher gal-3 concentrations (*p* < 0.05).

Table 3 presents SCORE medians in subgroups determined based on serum gal-3 concentration. The SCORE value was elevated in the group with higher gal-3 concentrations ≥221.31 ng/mL (median), *p* < 0.05.

**Figure 1:**
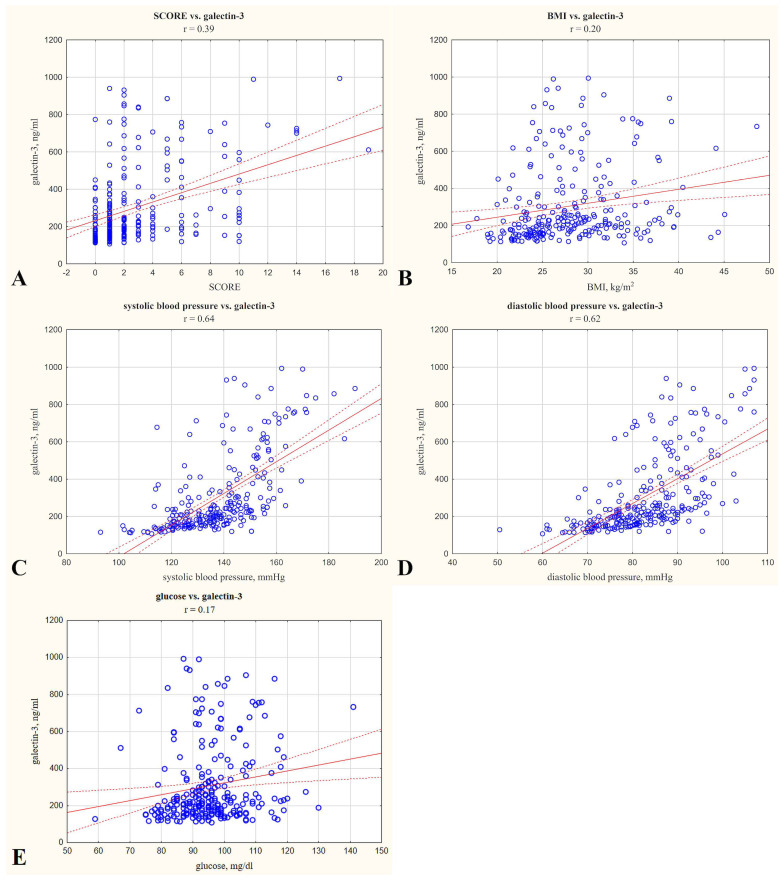
Scatter plots showing the correlations between galectin-3 concentration in serum (ng/mL) and selected quantitative variables in the study group: SCORE (**A**); BMI (**B**); systolic blood pressure (systolic BP, (**C**)); diastolic blood pressure (diastolic BP, (**D**)); glucose concentration (**E**). Each plot includes a regression line with a confidence interval and a Sperman correlation coefficient (r) to illustrate the strength of the relationship.

The mean BMI was 28.06 (5.46) kg/m^2^, which falls within the overweight category. Galectin-3 concentration demonstrated significant (*p* < 0.05) positive correlations with glucose concentrations (r = 0.17), BMI (r = 0.20), SCORE (r = 0.39), and systolic and diastolic BP (r = 0.64 and r = 0.62, respectively). Correlations with r < 0.4 are considered weak, indicating a limited strength of association for BMI, glucose concentrations, and SCORE [12]. Gal-3 did not correlate statistically significantly with total cholesterol, age, LDL, HDL, or triglycerides. These correlations are illustrated in Figure 1, which presents the relationships between gal-3 concentrations and selected quantitative variables in the study group.

The SCORE vs. galectin-3 concentration relationship was assessed in a backward stepwise multivariable regression analysis. Gal-3 concentration was considered the dependent variable. SCORE and the remaining clinical variables assessed were considered as potential independent variables. Due to multicollinearity, variables included in the SCORE scale were excluded from the potential independent variables. It was shown that elevated SCORE and higher diastolic BP are independent risk factors for increased serum gal-3 concentrations. The results of the regression analysis are shown in Table 4.

In assessing the statistical power of the obtained regression model, an assessment of the percentage of variance explained by the obtained regression model in the studied group was used. The obtained regression model explains 79.5% of the variance in the studied group.

**Figure 2:**
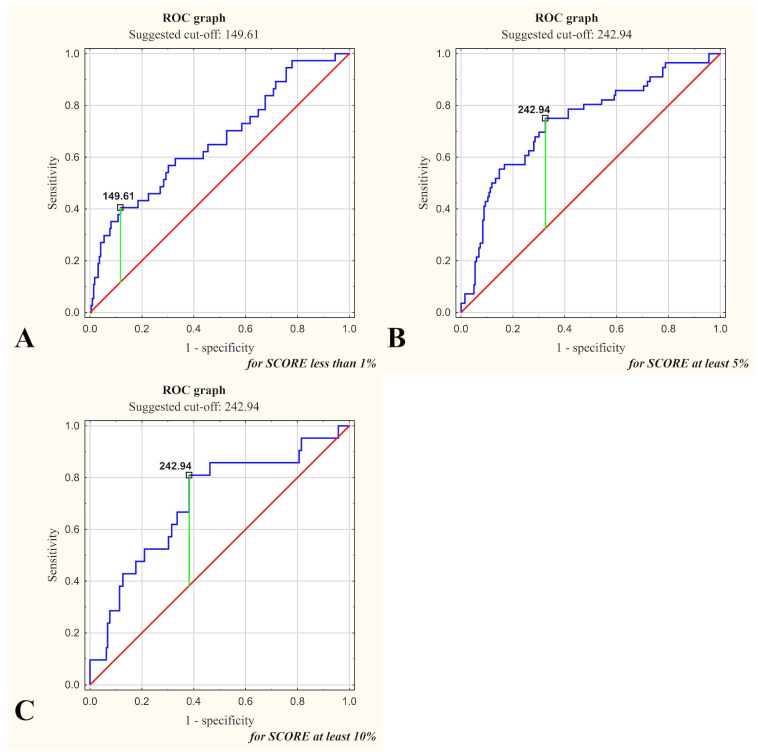
ROC curves for serum galectin-3 concentration illustrating its diagnostic utility in predicting SCORE categories ((**A**) <1%, (**B**) ≥5%, and (**C**) ≥10%). The suggested cut-off values are indicated on the graphs, with sensitivity and specificity detailed for each threshold. For example, for SCORE ≥ 10%, the optimal cut-off was 242.94 ng/mL (81.0% specificity; 61.8% sensitivity). The blue line represents the ROC curve, the red diagonal shows the reference line for a non-discriminative test (line of no discrimination), and the green vertical line indicates the optimal cut-off point (Youden index).

Galectin-3 concentration as a predictor for SCORE < 1% demonstrated a threshold value of <149.61 ng/mL (specificity, 0.378; sensitivity 0.883). For SCORE ≥ 5%, the threshold value reached ≥242.94 ng/mL (specificity, 0.750; sensitivity 0.675). For SCORE ≥ 10%, the same threshold value of ≥242.94 ng/mL was reached (specificity, 0.810; sensitivity 0.618). The highest classification accuracy (0.811) was achieved for SCORE < 1%. The LR+ and LR− values indicate the moderate diagnostic utility of gal-3 concentration in predicting SCORE. These results are detailed in Table 5 and illustrated with ROC curves in Figure 2.

The analysis of the relationship of gal-3 concentrations by gender and additionally by age, tobacco smoking status, systolic BP, and total cholesterol showed significant differences between subgroups (Table 6). Among males and females, higher concentrations of gal-3 were observed in older subjects (*p* < 0.05), smokers (*p* < 0.05), and those with higher systolic BP (*p* < 0.05). In the analysis including total cholesterol, significantly increased gal-3 concentrations were obtained only in the group of females with higher cholesterol levels (*p* < 0.05), while in males, the differences did not reach statistical significance.

## 3. Discussion

Gal-3 has been identified as a key biomarker associated with inflammatory and fibrotic mechanisms contributing to CVD. In this study, increased gal-3 concentrations correlated positively significantly with elevated SCORE values and clinical markers, such as blood pressure, BMI, and glucose concentrations. Our study supports the findings of other studies linking gal-3 to an increased risk of HF, atherosclerosis progression, and cardiovascular complications [13,14]. We also observed that gal-3 concentrations increased with age, consistent with Xue et al. [15] and Sanchis-Gomar et al. [16], who suggested this trend is due to cumulative inflammatory and fibrotic changes over time. Moreover, smokers in our cohort had higher gal-3 concentrations, consistent with Sharma et al. [17], highlighting tobacco-related toxins as triggers for gal-3-mediated inflammatory pathways. These findings suggest the biomarker’s utility in identifying high-risk patients with specific risk factors, such as smoking. Gal-3 highly correlates with blood pressure. This agrees with the data of Schwerg et al. [18] and M. Zeytinli Aksit et al. [19], who found positive correlations with BMI and arterial hypertension. However, while gal-3 showed robust associations with blood pressure and glucose, no significant correlations were observed between gal-3 concentrations and lipid parameters (total cholesterol or LDL cholesterol) in our study. This aligns with suggestions that gal-3 reflects pathways beyond traditional lipid-centric models, potentially related to microvascular dysfunction rather than macrovascular processes. However, Święcki et al. [20] concluded that patients with hyperlipidemia had elevated galectin-3 concentrations, which decreased during statin therapy. This suggests a potential link between gal-3 and lipid metabolism. While our study did not replicate this observation, it highlights a possible link between gal-3 and lipid metabolism that warrants further investigation.

In our study, SCORE was the primary tool for assessing 10-year cardiovascular mortality risk in the European population. Although the Framingham Risk Score (FRS) remains widely applied, particularly in North America for nonfatal events [21], SCORE aligns more closely with European guidelines and fatality prevention. This justifies its use in our cohort, where the majority of participants fell into the SCORE 1–4% category, indicating moderate risk. Galectin-3 concentrations were significantly higher in subgroups with SCORE ≥ 5%, supporting its potential utility in stratifying patients within higher-risk categories.

Our analysis identified a gal-3 threshold of <149.61 ng/mL, which demonstrated high sensitivity (88.3%) but low specificity (37.8%) for identifying patients with SCORE < 1%. We initially also examined gal-3’s predictive capacity for identifying low cardiovascular risk (SCORE < 1%), hypothesizing its potential function as a ‘rule-out’ biomarker. However, due to its low specificity (37.8%), the clinical usefulness of gal-3 in this context appears limited. Clinically, it seems more relevant to focus on gal-3’s predictive value for higher cardiovascular risk thresholds (SCORE ≥ 5% and ≥10%), where earlier preventive interventions might significantly alter clinical outcomes. Diagnostic performance was moderate for higher-risk categories (≥5% or ≥10%), suggesting the need for further research to refine clinical applications. Elevated gal-3 concentrations may complement standard risk models, particularly in intermediate-risk groups (5–9%), where they could support earlier interventions, advanced imaging, or lifestyle modifications.

Our findings show that gal-3 correlates with cardiovascular risk, as measured by the SCORE scale. Our regression model showed that higher SCORE scale and higher diastolic BP values were independent predictors of elevated gal-3 concentrations, suggesting a potential link between this biomarker and overall CVD risk and pointing to the need to take these parameters into account in the clinical evaluation of patients. Further studies are necessary to establish its predictive utility and clinical applications. Causal interpretations are obviously limited due to the cross-sectional nature of our study. We recognize that the small sample size, particularly in higher SCORE categories, limits the precision of our estimates. This is an important limitation that future research should address.

Gal-3 appears more closely linked to inflammatory and fibrotic pathways, particularly in hypertension, glycemic control, and smoking, while its association with lipid parameters was not apparent in our study group. These findings align with studies that identified galectin-3 is a promising therapeutic target in CVD [22,23].

The main strengths of our study include using validated risk assessment tools (e.g., SCORE scale) in combination with gal-3, a biomarker reflecting inflammatory and fibrotic processes. By analyzing a well-characterized Polish subpopulation within the PURE study, we provide insights into cardiovascular risk stratification in a region with a distinct epidemiological profile, where hypertension and smoking are more prevalent compared to Western Europe. This regional perspective is particularly relevant given the limited biomarker-based risk assessment data available for Central and Eastern European populations.

One of the limitations of our study is its focus on a single biomarker without incorporating additional cardiovascular risk markers, such as renal function parameters (e.g., creatinine) or echocardiographic indices (e.g., left ventricular EF), which could provide a more comprehensive assessment of cardiovascular risk. Second, while our study design allowed for cross-sectional correlations, it does not provide longitudinal data on cardiovascular events, limiting causal inferences. Third, although gal-3 was analyzed as a continuous variable, the lack of well-defined cut-off values limits its direct clinical applicability, emphasizing the need for further validation in larger cohorts. Moreover, the moderate sensitivity and specificity values obtained in our ROC analyses underscore the limitations in the diagnostic accuracy of gal-3 as a single predictive biomarker. Consequently, its clinical utility should be carefully interpreted, highlighting the potential necessity of combining galectin-3 with other biomarkers or clinical parameters to enhance predictive precision. Another important limitation is related to the relatively small subgroup sizes, especially in the highest-risk SCORE categories, which could affect statistical power and the accuracy of our estimates. Therefore, larger-scale studies and multicenter cohorts are warranted to verify our findings and further explore potential regional differences. Our study did not include measurements of direct vascular parameters (e.g., arterial stiffness, or intima-media thickness of carotid arteries), which could better reflect the vascular pathology associated with gal-3 concentrations. The aforementioned measurements could further clarify the pathophysiological significance of gal-3 in future CVD risk stratification. Finally, statistical analyses were performed under the assumption of normal distribution for key variables, which was verified before analysis; however, future studies should consider alternative statistical approaches, including nonparametric models, to confirm the reliability of our study.

Future studies should include longitudinal designs, larger study groups, and comparisons of multiple risk scales, such as SCORE2 or FRS. Further validation of gal-3 thresholds and the assessment of their cost-effectiveness in primary and secondary prevention settings are also necessary. Additionally, it remains to be determined whether interventions aimed at lowering gal-3 concentrations through anti-inflammatory or antifibrotic treatments could improve cardiovascular outcomes, which should be addressed in future longitudinal research. Future research should also utilize the updated SCORE2 algorithm to provide a contemporary CVD risk evaluation. There is a need for studies examining whether elevated galectin-3 levels can help identify individuals classified as low- or moderate-risk according to SCORE (or the newer SCORE2) who may nevertheless benefit from intensified monitoring and prevention strategies.

Our study highlights the possible value of gal-3 as an adjunct biomarker in cardiovascular risk assessment, particularly in populations with distinct risk factor distributions, such as the Polish subpopulation of the PURE study. While our findings align with previous research on the role of serum gal-3 in CVD risk, they also provide new insights into its application in SCORE-based risk stratification within a Central European cohort. Given regional differences in cardiovascular risk profiles, these results contribute to the ongoing discussion on the utility of gal-3 as a complementary marker beyond traditional lipid-based models.

Recently, increasing attention has been given to the role of genetic mutations, such as the JAK2V617F mutation, in augmenting cardiovascular risk. The JAK2 mutation contributes to elevated cardiovascular risk through enhanced chronic inflammation, oxidative stress, and endothelial dysfunction. Specifically, JAK2-mutated endothelial cells exhibit an increased expression of adhesion molecules, promoting abnormal leukocyte and platelet interactions, thereby facilitating thrombosis and vascular injury. Additionally, JAK2V617F-positive neutrophils display heightened activation, generating excessive neutrophil extracellular traps, which further exacerbate thrombogenicity and atherosclerotic plaque instability [24]. Given these insights, integrating genetic markers like JAK2 into cardiovascular risk assessment models alongside biomarkers such as gal-3 could improve the early identification of high-risk individuals, particularly within populations harboring both inflammatory and prothrombotic profiles.

While previous research has explored the relationship between galectin-3 and cardiovascular outcomes, this study focuses on its potential role in primary risk assessment using SCORE in a Central European cohort. Given the clinical relevance of identifying high-risk individuals early, these findings warrant further validation in larger prospective studies. Despite being a single-biomarker study, our findings suggest that galectin-3 may provide additional risk stratification value when used alongside established cardiovascular risk scores. Further studies incorporating multimodal biomarker panels and clinical outcomes are warranted to validate its predictive utility.

## 4. Materials and Methods

We performed our cross-sectional study on a selected subgroup of participants from the PURE cohort, conducted by the Population Health Research Institute, McMaster University, Hamilton, Ontario, Canada. This project has been ongoing in one of our units since 2007.

**Figure 3:**
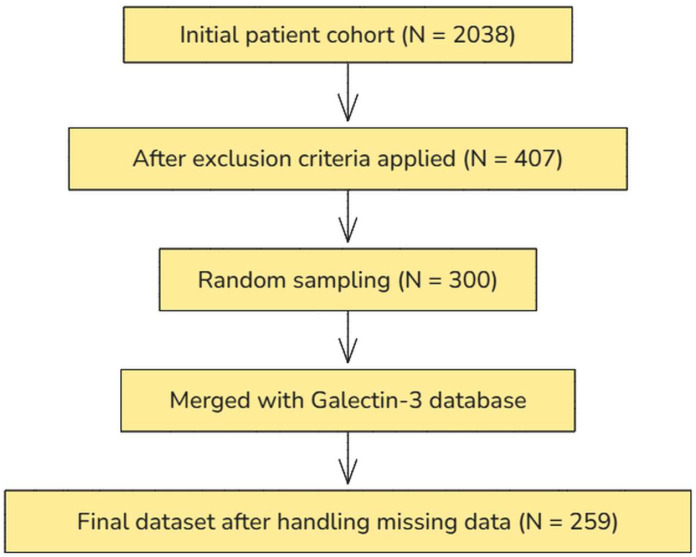
Flowchart illustrating the stepwise selection process from the initial cohort of 2038 patients to the final analytical dataset of 259 patients.

The initial dataset comprised 2038 patients. The selection process was conducted in four sequential steps. First, predefined exclusion criteria were applied, resulting in a subset of 407 eligible patients. A random sample of 300 patients was chosen (using a computer-generated randomization procedure) to ensure a balanced dataset with complete information while minimizing potential biases related to missing data in certain variables. This approach also maintained statistical efficiency while allowing for feasible biochemical analyses within the available resources. The selected sample was subsequently merged with an external database containing gal-3 measurements. Finally, data completeness was assessed, and observations with missing values were excluded, yielding a final analytical dataset of 259 patients. The stepwise selection process is shown in Figure 3. The inclusion criteria for this analysis were age above 40 years and complete data on tobacco smoking status, blood pressure, and total cholesterol. Individuals with a previous diagnosis of stroke, diabetes, myocardial infarction, or renal failure—either at baseline or during the follow-up assessments at 3, 6, and 9 years—were excluded from the analyses.

Clinical risk assessment using the SCORE system was performed based on data collected at the three-year observation. The use of the SCORE scale was dictated by the fact that data have been collected on patients since 2007. Blood samples for serum gal-3 assessment were obtained at the same time point, ensuring that both assessments were conducted simultaneously.

This study utilized two tools: the SCORE scale and measurements of gal-3 concentrations in serum. Galectin-3 concentrations were determined in serum samples using an ELISA kit (BT LAB, Jiaxing, Zhejiang, China; Cat. No. E1951Hu) according to the provided instructions. The study population was divided into subgroups for comparisons based on the criteria presented in Table 2 and Table 3. In Table 2, participants were categorized by age based on the median (≥56 and <56 years), gender, smoking status (no/yes), total cholesterol levels based on the median (≥181 mg/dL and <181 mg/dL), systolic blood pressure values based on the median (≥137.0 mmHg and <137.0 mmHg), and SCORE categories (<1%, 1–4%, 5–9%, and ≥10%). Table 3 defines subgroups based on the median galectin-3 concentration (<221.31 ng/mL and ≥221.31 ng/mL) in relation to SCORE values (%). Statistical comparisons were conducted to check the relationships between gal-3 concentrations, cardiovascular risk (SCORE), and other clinical and demographic variables.

We analyzed categorical variables using Fisher’s exact test (when expected cell counts < 5) and the chi-square test. Comparisons between subgroups defined by SCORE and gal-3 concentrations were analyzed using the parametric Student’s *t*-test or its nonparametric equivalents when the assumption of normal distribution was not met. We considered a *p* < 0.05 (α = 5%) value as statistically significant. The normality of continuous variables, including SCORE and galectin-3 concentrations, was verified prior to statistical analysis. If at least one of the variables did not have a normal distribution, then we used nonparametric Sperman correlations for analysis. Sperman’s correlation coefficient evaluated the relationship between gal-3 concentrations (not normally distributed in our study) and quantitative variables, which were further illustrated with scatter plots. ROC curves were used to assess the predictive ability of gal-3 for different SCORE categories. Specificity, sensitivity, predictive values, and likelihood ratios (LR+ and LR−) were analyzed, and the optimal cut-off points were established based on the Youden index. Additionally, classification accuracy was computed as a measure of diagnostic performance. This comprehensive analytical approach enabled a multifaceted evaluation of galectin-3’s role as a cardiovascular risk biomarker and its association with other clinical factors.

Post hoc power analysis was also conducted, instead of calculating the required sample size. We wanted to verify that the study had sufficient power to detect the observed relationships. The power for the main correlation analysis was calculated and confirmed to be adequate for detecting meaningful relationships. The post hoc sample size calculation, based on an observed effect size (r = 0.39), indicated that 79 participants were required to achieve 95% power at α = 0.05. The study cohort of 259 patients exceeded this threshold. We used the R software (version 4.4.2, pwr package) for our calculations. For all other statistical analyses, we used Statistica software (version 14.0.0.15).

The Bioethics Committee of the Wroclaw Medical University gave a positive opinion on our study. Written informed consent was obtained from each study participant in accordance with the ethical standards of the 1964 Declaration of Helsinki.

## 5. Conclusions

Taken together, gal-3 concentrations show a significant association with cardiovascular risk as estimated by the SCORE scale. However, the role of gal-3 as a predictive criterion for high cardiovascular risk (≥5% or ≥10%) requires further validation in studies with larger cohorts and clinical follow-up data. While the threshold value of ≥242.94 ng/mL identified in our study may provide a basis for future research, limited specificity regarding low-risk categories (<1%) highlights the need for additional studies to optimize the clinical application of gal-3.

## Figures and Tables

**Table 1:** Quantitative and qualitative characterization of the patient cohort (*n* = 259).

Quantitative Variables					
Variable	Mean	Median	Q1	Q3	SD
Age [years]	55.54	56.00	49.00	61.00	8.00
BMI [kg/m^2^]	28.06	27.24	24.07	31.26	5.46
SBP [mmHg]	137.91	137.00	126.50	148.50	15.91
DBP [mmHg]	82.73	82.50	76.50	89.00	9.80
Total cholesterol [mg/dL]	175.93	181.00	166.00	190.00	18.64
Triglycerides [mg/dL]	103.40	91.00	70.00	119.00	54.57
LDL cholesterol [mg/dL]	100.95	102.00	88.00	113.00	18.18
HDL cholesterol [mg/dL]	54.70	53.00	44.00	64.00	14.02
Glucose [mg/dL]	95.56	95.00	88.00	101.00	11.10
Galectin-3 [ng/mL]	305.38	221.32	161.64	360.00	209.84
SCORE	2.96	2.00	1.00	4.00	3.33
**Qualitative Variables**					
**Variable**	**Absolute Value**		**Percentage**		
Male gender	109		42.1%		
Female gender	150		57.9%		
Smoking	37		14.3%		
SCORE < 1%	37		14.3%		
SCORE 1–4%	167		64.5%		
SCORE 5–9%	34		13.1%		
SCORE ≥ 10%	21		8.1%		

Abbreviations: BMI, body mass index; DBP, diastolic blood pressure; HDL, high-density lipoprotein; LDL, low-density lipoprotein; SBP, systolic blood pressure; SD, standard deviation; SCORE, Systematic Coronary Risk Evaluation; Q1, first quartile (25th percentile); Q3, third quartile (75th percentile).

**Table 2:** Serum galectin-3 concentration in subgroups.

Variable		Galectin-3 [ng/mL]		
		Median	Q1	Q3
Age ^a^	≥Me (≥56 years)	228.09	177.44	456.33
	<Me (<56 years)	209.13	152.06	301.90
	*p*	*p* < 0.05		
Gender	male	247.31	191.71	448.66
	female	199.73	154.33	313.41
	*p*	*p* < 0.05		
Smoking	yes	410.00	237.20	709.11
	no	206.27	156.19	302.54
	*p*	*p* < 0.05		
SBP ^a^	≥Me (≥137.0 mmHg)	302.35	221.38	584.75
	<Me (<137.00 mmHg)	170.67	144.82	213.01
	*p*	*p* < 0.05		
Total cholesterol ^a^	≥Me (≥181 mg/dL)	229.17	162.45	410.93
	<Me (<181 mg/dL)	208.90	160.94	302.54
	*p*	not statistically significant		
SCORE	<1%	177.16	130.12	259.28
	1–4%	206.42	161.64	299.69
	5–9%	450.05	207.72	639.24
	≥10%	382.99	243.00	699.89
	*p*	*p* < 0.05 for: <1% vs. 5–9%, <1% vs. ≥10%, 1–4% vs. 5–9%, 1–4% vs. ≥10%		
	<1%	177.16	130.12	259.28
	≥1%	225.61	168.35	389.43
	*p*	*p* < 0.05		
	<5%	205.63	155.19	282.75
	≥5%	429.80	232.98	653.63
	*p*	*p* < 0.05		
	<10%	209.22	160.24	337.33
	≥10%	382.99	243.00	699.89
	*p*	*p* < 0.05		

^a^ division into two subgroups based on the median (Me) of a given parameter. Abbreviations: Me, median; SBP, systolic blood pressure; SCORE, Systematic Coronary Risk Evaluation.

**Table 3:** SCORE in subgroups determined based on the serum galectin-3 concentration.

Variable		SCORE [%]		
		Median	Q1	Q3
Galectin-3 ^a^	<Me (<221.31 ng/mL)	1.00	1.00	2.00
	≥Me (≥221.31 ng/mL)	2.00	1.00	6.00
	*p* ^b^	<0.05		

^a^ division into two subgroups based on the median (Me) of a given parameter; ^b^ Sperman’s nonparametric correlations. Abbreviations: Me, median; SCORE, Systematic Coronary Risk Evaluation; Q1, first quartile (25th percentile); Q3, third quartile (75th percentile).

**Table 4:** Estimation results for the model derived from the regression analysis.

Model for Galectin-3 [ng/mL]				
	Regression Coefficient	SEM of Rc	*p*	*p* of the Model
SCORE	22.443	3.305	<0.001	<0.01
DBP	7.537	0.878	<0.001	

Abbreviations: DBP, diastolic blood pressure; SEM of Rc, standard error of the mean of the regression coefficient.

**Table 5:** The specificity and sensitivity of serum galectin-3 concentration as a determinant of SCORE.

Variable	SCORE < 1%	SCORE ≥ 5%	SCORE ≥ 10%
Galectin-3 Concentration as Predictor of SCORE	<149.61 ng/mL	≥242.94 ng/mL	≥242.94 ng/mL
Sensitivity	0.883 ^a^	0.675	0.618
Specificity	0.378	0.750	0.810 ^a^
Accuracy	0.811 ^a^	0.691	0.633
LR+	1.420	2.700	3.243
LR−	0.310	0.433	0.472
PPV	0.895	0.907	0.974
NPV	0.350	0.389	0.157

^a^ highest prediction. Abbreviations: LR−, negative likelihood ratio; LR+, positive likelihood ratio; NPV, negative predictive values; PPV, positive predictive values; SCORE, Systematic Coronary Risk Evaluation.

**Table 6:** Galectin-3 concentrations by gender and additionally by selected cardiovascular risk factors (*n* = 259).

Variable			Galectin-3 [ng/mL]			*p*
Gender	Risk Factor	Subgroup	Median	Q1	Q3	
Female	Age ^a^	≥Me (≥56 years)	205.63	167.60	351.16	ns
		<Me (<56 years)	177.66	144.37	273.10	
Male		≥Me (≥56 years)	259.65	199.38	575.68	*p* < 0.05
		<Me (<56 years)	228.12	174.85	369.51	
Female	Smoking	yes	301.90	237.08	677.43	*p* < 0.05
		no	187.32	150.20	279.13	
Male		yes	527.29	274.83	727.84	*p* < 0.05
		no	238.24	176.63	354.63	
Female	SBP ^a^	≥Me (≥137 mmHg)	332.25	214.78	566.94	*p* < 0.05
		<Me (<137 mmHg)	166.55	140.15	566.94	
Male		≥Me (≥137 mmHg)	278.36	221.45	593.82	*p* < 0.05
		<Me (<137 mmHg)	190.79	153.25	256.22	
Female	Total cholesterol ^a^	≥Me (≥181 mg/dL)	209.11	156.84	376.89	*p* < 0.05
		<Me (<181 mg/dL)	182.71	149.86	249.29	
Male		≥Me (≥181 mg/dL)	251.76	191.71	516.90	ns
		<Me (<181 mg/dL)	243.00	190.37	448.66	

^a^ division into two subgroups based on the median (Me) of a given parameter. Abbreviations: Me, median; ns, not statistically significant; SBP, systolic blood pressure; Q1, first quartile (25th percentile); Q3, third quartile (75th percentile).

## Data Availability

The data presented in this study are available on request from the corresponding author (the data are not publicly available due to privacy or ethical restrictions).

## Abbreviations

The following abbreviations are used in this manuscript:

AH	Arterial hypertension
BMI	Body mass index
BP	Blood pressure
CVD	Cardiovascular diseases
EF	Ejection fraction
FDA	Food and Drug Administration
FRS	Framingham Risk Score
DBP	Diastolic blood pressure
Gal-3	Galectin-3
HDL	High-density lipoprotein
HF	Heart failure
HFpEF	Heart failure with preserved ejection fraction
HR	Hazard ratio
LDL	Low-density lipoprotein
LR−	Negative likelihood ratio
LR+	Positive likelihood ratio
Me	Median
NPV	Negative predictive value
PPV	Positive predictive value
PURE	Prospective Urban and Rural Epidemiological Study
ROC	Receiver operating characteristic
SBP	Systolic blood pressure
SCORE	Systematic Coronary Risk Evaluation
SD	Standard deviation
Q1	first quartile (25th percentile)
Q3	third quartile (75th percentile)
